# Role of Borneol Induced Autophagy in Enhancing Radiosensitivity of Malignant Glioma

**DOI:** 10.3389/fonc.2021.749987

**Published:** 2021-11-30

**Authors:** Qinglin Li, Liang Xia, Caixing Sun, Huangjie Zhang, Mengying Zheng, Hongyan Zhang, Hongyang Lu, Zeng Wang

**Affiliations:** ^1^ Department of Scientific Research, The Cancer Hospital of the University of Chinese Academy of Sciences (Zhejiang Cancer Hospital), Institute of Basic Medicine and Cancer (IBMC), Chinese Academy of Sciences, Hangzhou, China; ^2^ Department of Neurotumor Surgery, The Cancer Hospital of the University of Chinese Academy of Sciences (Zhejiang Cancer Hospital), Institute of Basic Medicine and Cancer (IBMC), Chinese Academy of Sciences, Hangzhou, China; ^3^ Department of Pharmacy, The Cancer Hospital of the University of Chinese Academy of Sciences (Zhejiang Cancer Hospital), Institute of Basic Medicine and Cancer (IBMC), Chinese Academy of Sciences, Hangzhou, China; ^4^ Department of Medical School, Zhejiang University City College, Hangzhou, China; ^5^ Department of Thoracic Oncology, The Cancer Hospital of the University of Chinese Academy of Sciences (Zhejiang Cancer Hospital), Institute of Basic Medicine and Cancer (IBMC), Chinese Academy of Sciences, Hangzhou, China

**Keywords:** natural product, borneol, malignant glioma, autophagy, HIF-1α

## Abstract

Glioma is the common primary craniocerebral malignancy with unfavorable prognosis. It is currently treated by surgical resection supplemented by radiotherapy, although the resistance of glioma cells to radiation limits the therapeutic outcomes. The aim of the present study was to determine the potential radiosensitizing effects of borneol and the underlying mechanisms. We found that borneol administration along with radiotherapy significantly inhibited the growth of primary glioma cells *in vitro* and *in vivo*. Furthermore, borneol markedly increased the number of autophagosomes in the glioma cells, which coincided with increased expression of beclin-1 and LC3. And the combination of borneol and radiation exposure significantly decreased the expression levels of HIF-1α, mTORC1 and eIF4E. In addition, silencing mTORC1 and eIF4E upregulated Beclin-1 and LC3 and decreased the expression of HIF-1α, thereby inhibiting tumor cell proliferation. Our findings suggest that borneol sensitizes glioma cells to radiation by inducing autophagy *via* inhibition of the mTORC1/eIF4E/HIF-1α regulatory axis.

## 1 Introduction

Glioma accounts for about 60% of all brain tumors and is the most common primary craniocerebral malignancy originating from glial cells of the brain and spinal cord (1–3). Currently, gliomas are classified by the World Health Organization (WHO) into low and high-grade neurogliomas (4). Glioblastoma (GBM) is the most aggressive and common form of adult brain cancer, and is characterized by high recurrence and mortality, and low cure rates (5). In addition, most malignant gliomas infiltrate into the surrounding tissues resulting in lack of clearly demarcated boundaries. The average survival duration of GBM patients is only 14 months, and the five-year mortality rate exceeds 95% (6–8). Currently, surgical resection, radiotherapy and adjuvant chemotherapy are the primary treatment strategies for GBM, although radiation tolerance often leads to tumor recurrence.

Hypoxia-inducible factor-1α (HIF-1α) is activated in the hypoxic tumor microenvironment and promotes the transcription of genes associated with tumor invasion, proliferation and radio-resistance (9–13). The tumor cells often develop resistance to hypoxia *via* autophagy, a lysosome-dependent mechanism of degrading damaged proteins and organelles that ensures nutrient recycling, protein synthesis, cellular homeostasis and survival during stress (14–16). Autophagy is initiated with the formation of a “bubble” of lipid bilayer around the proteins/organelles, which is then elongated to vesicles known as autophagosomes in the presence of LC3 (17, 18). Beclin-1 also plays a crucial role in the induction of autophagy, and is an established tumor suppressor that inhibits the genesis and progression of tumors by promoting autophagy and apoptosis. It is aberrantly expressed in various tumors, and is regulated by HIF-1α. Studies show that Beclin-1 downregulation and HIF-1α overexpression are conducive to tumor progression and metastasis, and therefore portend poor prognosis (19, 20).

The mammalian target of rapamycin (mTOR) pathway is the key regulator of autophagy, and is activated in response to growth factors, metabolic stress, low energy levels, hypoxia and nutrient deficiency (21–23). In a previous study, we found that the natural terpene derivative borneol inhibited HIF-1α in primary glioma cells by regulating the mTORC1/eIF4E pathway, and induced autophagy and apoptosis. The aim of the present study was to evaluate the potential radio-sensitizing effect of borneol on glioma cells and explore the role of autophagy.

## 2 Experimental Methods

### 2.1 Establishment of Rodent C6 Intracranial Tumor Transplantation Model and Treatment Regimen

SPF male SD rats were provided by Shanghai Sipur Bikai Experimental Animal Co. Ltd. After 2 weeks of acclimatization, the animals were divided into the control, radiotherapy, borneol and combination therapy groups (n=6 each). Glioma cells were inoculated to establishment the tumor model as previously described (24). Two weeks later, the animals from the appropriate groups were anesthetized with pentobarbital and irradiated once with 15Gy dosage. Borneol (16 mg/kg) was administered for 7 days before and 3 days after radiotherapy. The general condition and survival of the animals were recorded, and the relative tumor volume was measured.

### 2.2 Immunohistochemistry (IHC)

The tumor tissues were fixed in formalin, dehydrated, embedded in paraffin and cut into ultra-thin sections. After rehydration and antigen retrieval, the sections were incubated overnight with the specific primary antibody at 4°C, followed by biotinylated secondary antibody. The sections were then washed with PBS, probed with avidin-horseradish peroxidase complex, developed with the DAB chromogenic agent, rinsed with water, and counterstained with hematoxylin. The stained sections were dehydrated, cleared with xylene, sealed with neutral gum, and observed under a light microscope. The positively stained cells were counted in 6 random non-overlapping fields at 10x-40x magnification.

### 2.3 Transmission Electron Microscopy

The glioma tissues were washed with 0.1mM phosphate buffer, fixed with 1% osmium acid for 2 hours, rinsed again with the same solution, and dehydrated through an alcohol gradient. After drying to critical point, the tissues were coated with platinum for 10 minutes, and observed by transmission electron microscopy (TEM).

### 2.4 Human Primary Glioma Cell Culture and CCK-8 Assay

Human primary glioma cells were isolated as previously described (24). The cells in the logarithmic growth phase were seeded into 96-well plates, and incubated for 24h. After irradiating with X-ray at the dose of 5Gy, the cells were cultured in the presence of varying concentrations of borneol (10, 20, 40 and 80µg/ml) for 48h. Untreated and non-irradiated controls were included. Ten microliters CCK-8 solution was added to each well and after a 2h incubation, the absorbance at 450nm was measured using a microplate analyzer. Each condition was tested in triplicates.

### 2.5 SiRNA/Gene Transfection

HIF-1α overexpression siRNA vectors were designed and synthesized as in our previous study (24), and the primary glioma cells were transfected with the respective constructs with Opti-MEM and Oligofectamine according to the manufacturer’s instructions. The stably transfected cells were irradiated (5Gy) and cultured with 20µg/ml borneol for 48h. Cell viability was evaluated as described in section *Human primary glioma cell culture and CCK-8 assay*.

### 2.6 Immunofluorescence and Western Blotting

The expression levels of HIF-1α, mTORC1, eIF4E, LC3 and Beclin1 proteins in the suitably transfected/treated cells were evaluated by immunofluorescence and western blotting with specific antibodies as previously described (24).

### 2.7 Statistical Analysis

SPSS22.0 was used for statistical analysis. All data were expressed as mean ± standard deviation (x ± s). T-test (homogeneity of variance) or Kruskal-Wallish test (heterogeneity of variance) was used to compare the means of two experimental groups. P<0.05 was considered statistically significant.

## 3 Results

### 3.1 The Combination of Borneol and Radiotherapy Inhibited Glioma Growth by Inducing Autophagy

**Figure 1:**
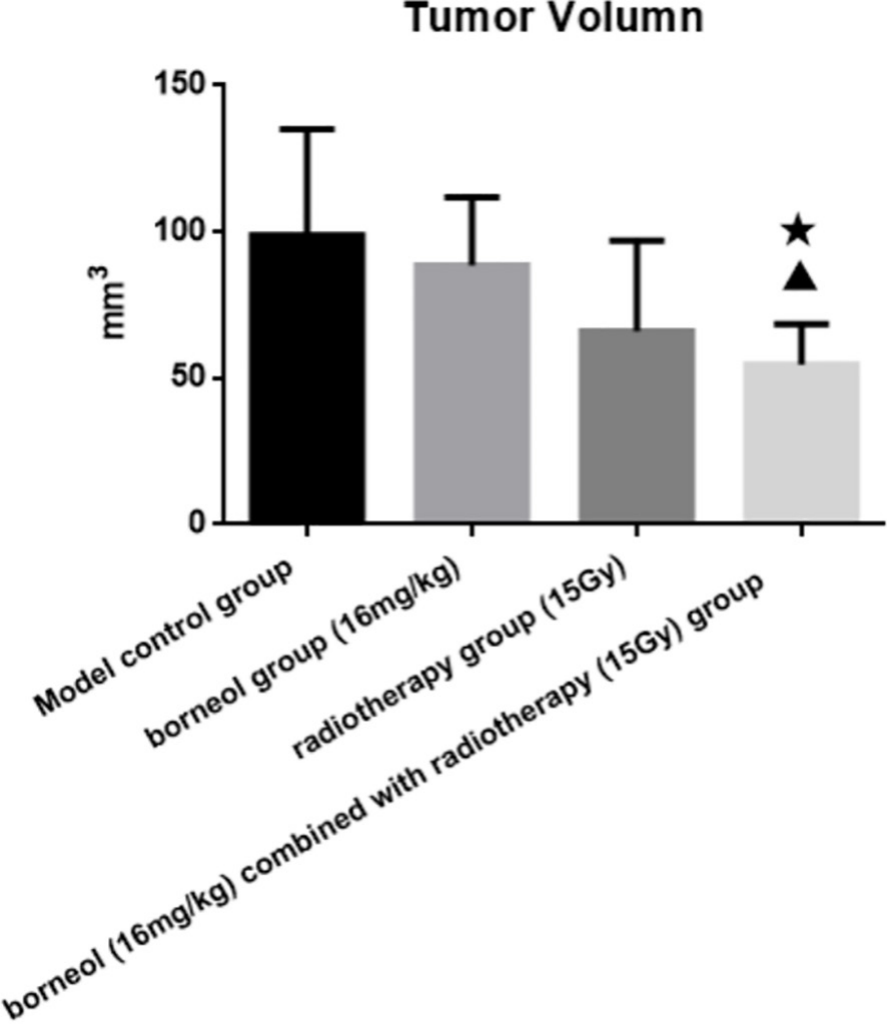
Tumor volume of brain glioma in rats after borneol and radiotherapy treatment( $\bar{x}$ ± S, n=6). Compared with the control group, ^▲^P<0.05; Compared with borneol group, ^★^P<0.05.

**Figure 2:**
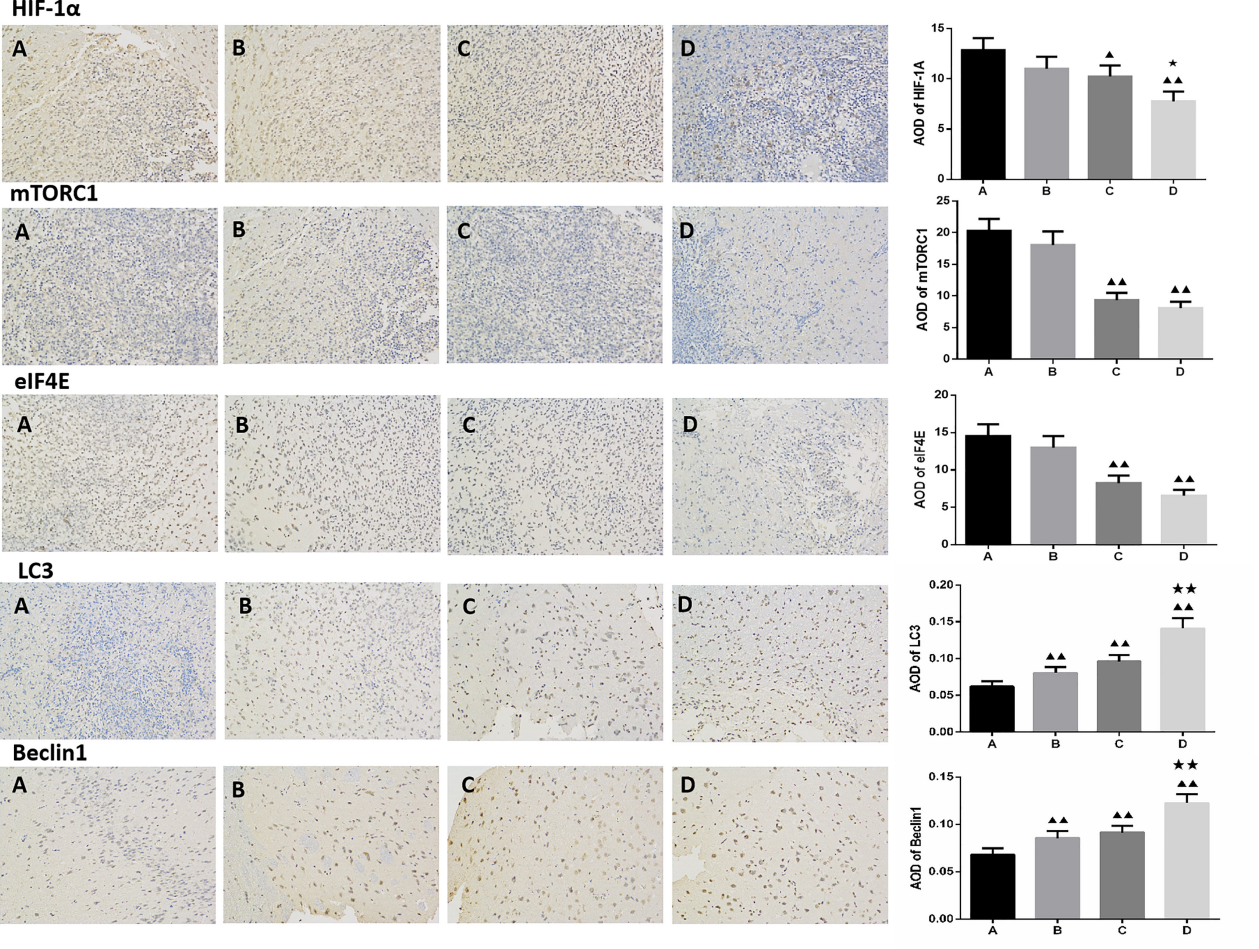
Immunohistochemical detection of HIF-1α, mTOR, eIF4E, LC3 and Beclin1 expression levels in glioma tissues treated by borneol and radiotherapy (n=3, ×200 times). **(A)** Control group **(B)** Borneol 16mg/Kg **(C)** Radiotherapy 15Gy group **(D)** Borneol 16mg/Kg combined radiotherapy 15Gy group. Compared with control group, ^▲^P<0.05, ^▲▲^P< 0.01; Compared with the radiation 5Gy group, ^★^P<0.05, ^★★^P< 0.01.

**Figure 3:**
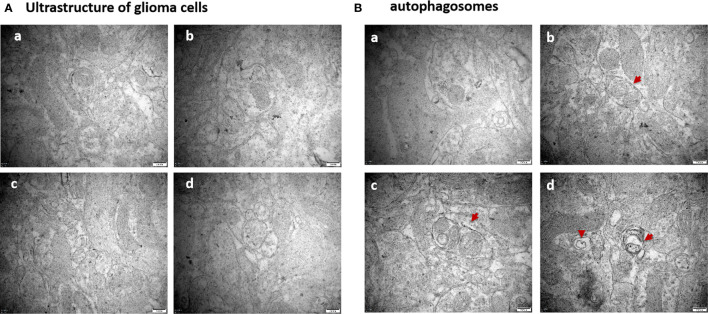
**(A)** Ultrastructure of glioma tissues observed by transmission electron microscopy (×60000 times); **(B)** Observation of the formation of autophagosomes in glioma tissues by transmission electron microscopy (×60000 times). The red arrows are autophagosomes. **(a)** Control group **(b)** Borneol 16mg/Kg **(c)** Radiotherapy 15Gy group **(d)** Borneol 16mg/Kg combined radiotherapy 15Gy group.

Compared to the untreated controls and the borneol group, borneol combined with radiotherapy group significantly decreased the tumor volume (P<0.05, P<0.05), and the tumor inhibition rate of the combination therapy was higher than the radiotherapy alone (**Figure 1**). As shown in **Figure 2**, the expression levels of HIF-1α, mTORC1 and eIF4E in glioma tissues was markedly lower in the animals treated with borneol and/or radiation compared to the untreated tumor-bearing group (P<0.05 or P<0.01). Furthermore, the different therapies increased the expression of LC3 and Beclin-1 in the gliomas (P<0.01). Consistent with this, TEM examination of the glioma tissues (**Figure 3A**) showed numerous autophagosomes in the borneol, radiotherapy and combination therapy groups compared to the untreated controls (**Figure 3B**). Taken together, borneol induced autophagy in the glioma cells and augmented radiation-mediated tumor inhibition.

### 3.2 Borneol Sensitized Glioma Cells to Radiation by Targeting the mTORC1/eIF4E/HIF-1α Pathway

**Figure 4:**
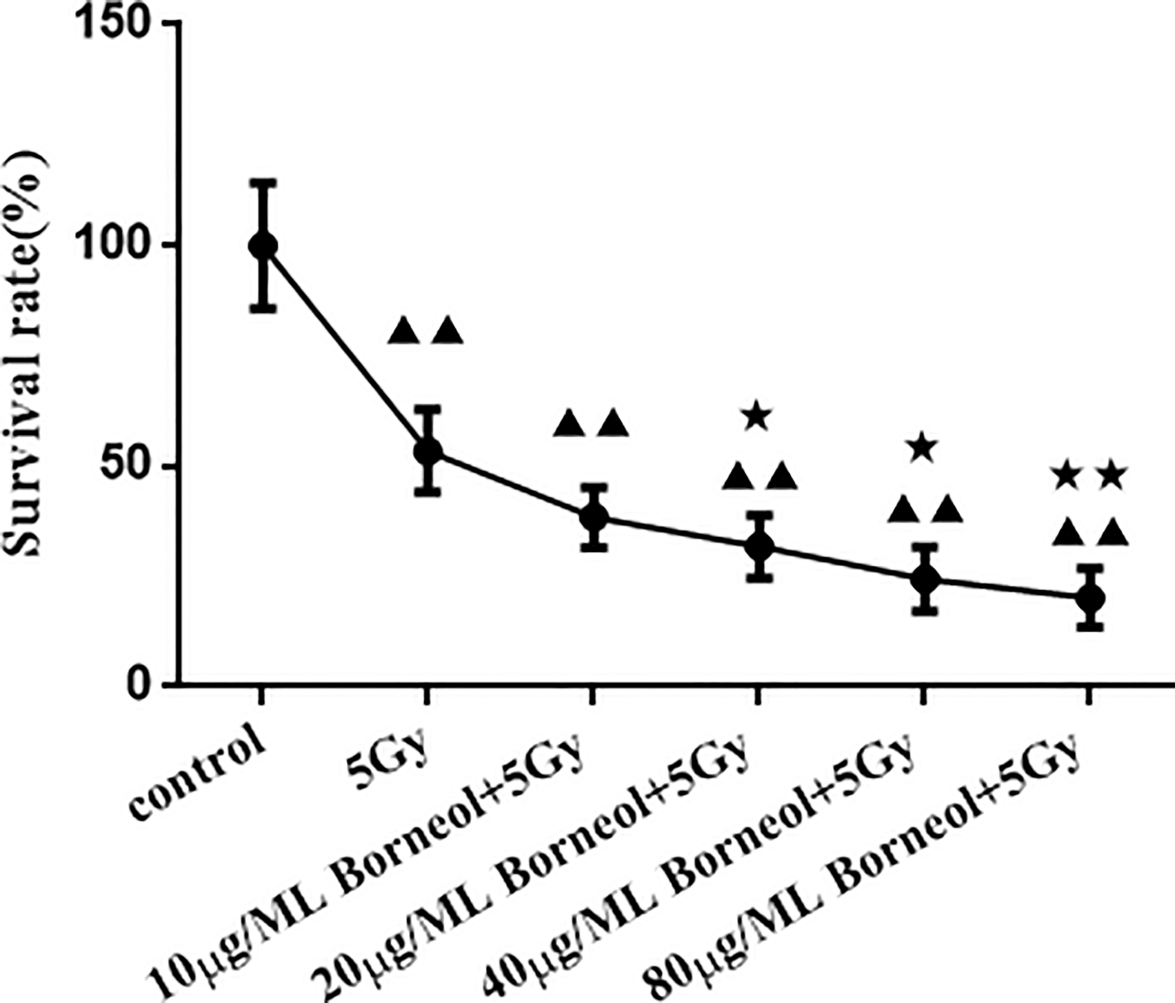
Effects of different concentrations of borneol on the growth of human glioma primary cultured cells under the radiation dose of 5Gy ( $\bar{x}$ ± S,*n*=3). Compared with control group, ^▲▲^P< 0.01; Compared with the radiation 5Gy group, ^★^P<0.05, ^★★^P< 0.01.

As shown in **Figure 4**, borneol significantly decreased the viability of the glioma cells *in vitro* in a dose-dependent manner when combined with 5Gy irradiation compared to cells subjected to either treatment (P < 0.05 for 20μg/ml and 40μg/ml borneol and p < 0.01 for 80μg/ml borneol).

**Figure 5:**
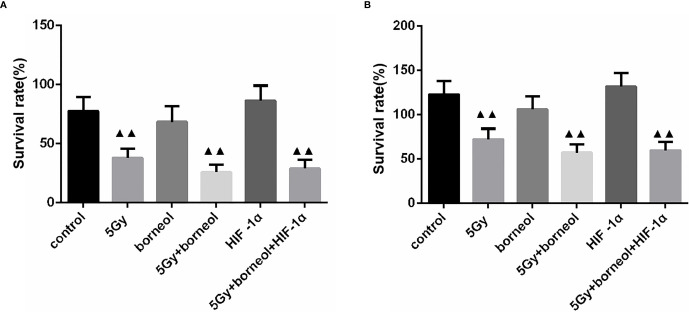
Cell growth Of borneol and radiotherapy treatment on HIF-1α silence **(A)** or overexpressing **(B)** cell ( $\bar{x}$ ± S,*n*=3). Compared with the control group, ^▲▲^P <0.01.

HIF-1α knockdown decreased the percentage of viable glioma cells, whereas ectopic expression of HIF-1α had the opposite effect (P > 0.05). However, the survival rate of the HIF-1α-overexpressing cells decreased significantly when treated with borneol and/or 5Gy radiation (P<0.05 or P<0.01), and the inhibitory effect of the combination treatment was greater. In the HIF-1α-silenced cells, borneol and radiotherapy further inhibited the proliferation rates (**Figure 5**).

**Figure 6:**
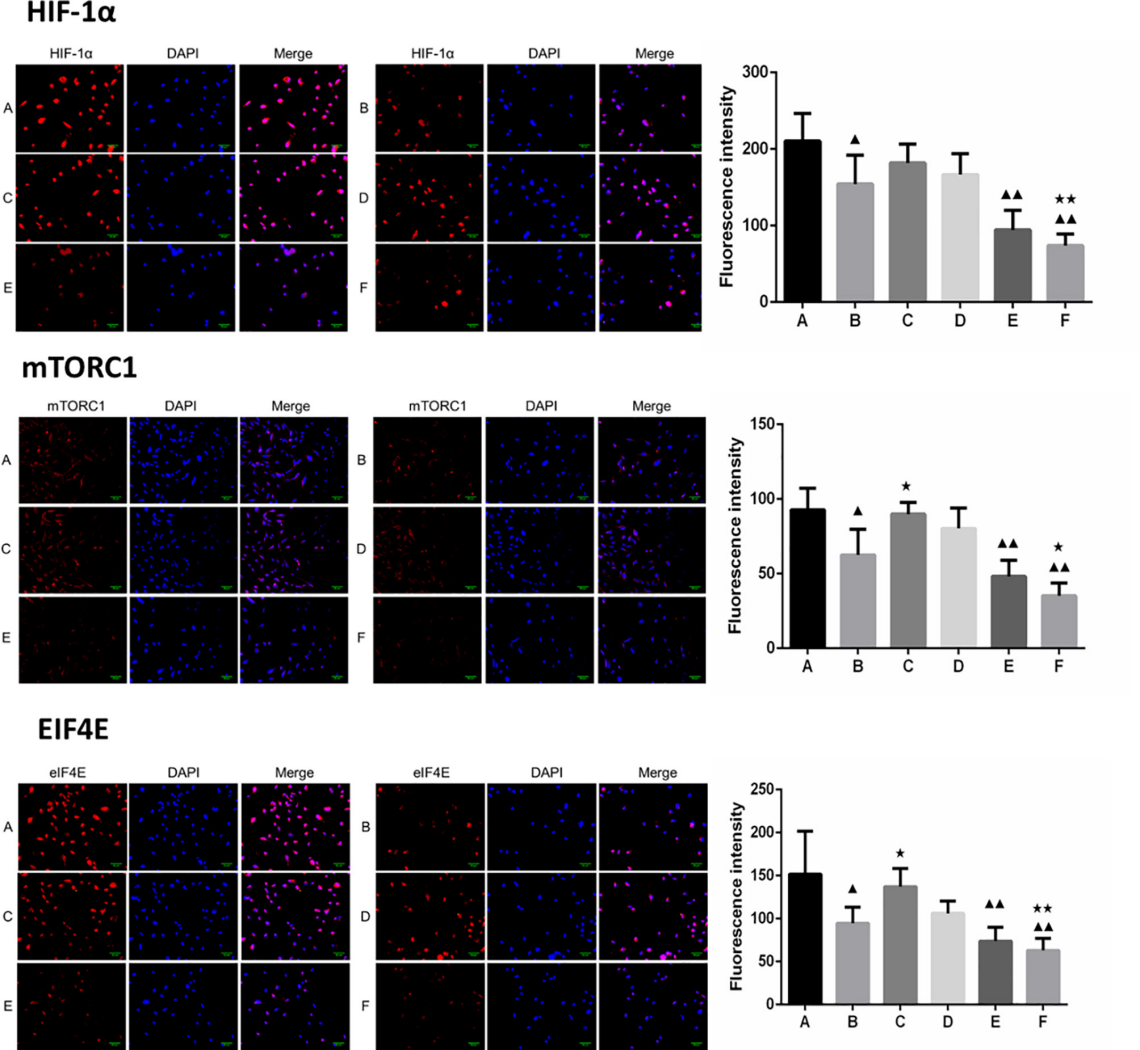
Expression of HIF-1α, mTORC1 and eIF4E in each group after borneol and radiotherapy treatment (200×) ( $\bar{x}$ ± S,*n*=5). **(A)** Control group **(B)** Radiotherapy 5Gy group **(C)** Borneol 20μg/ml 24h group **(D)** Borneol 20μg/ml 48h group **(E)** Borneol 20 μg/ml combined radiotherapy 5Gy group **(F)** Borneol 20μg/ml 48h combined radiotherapy 5Gy group. Compared with the control group, ^▲^P<0.05, ^▲▲^P< 0.01; Compared with radiotherapy group, ^★^P<0.05, ^★★^P< 0.01.

**Figure 7:**
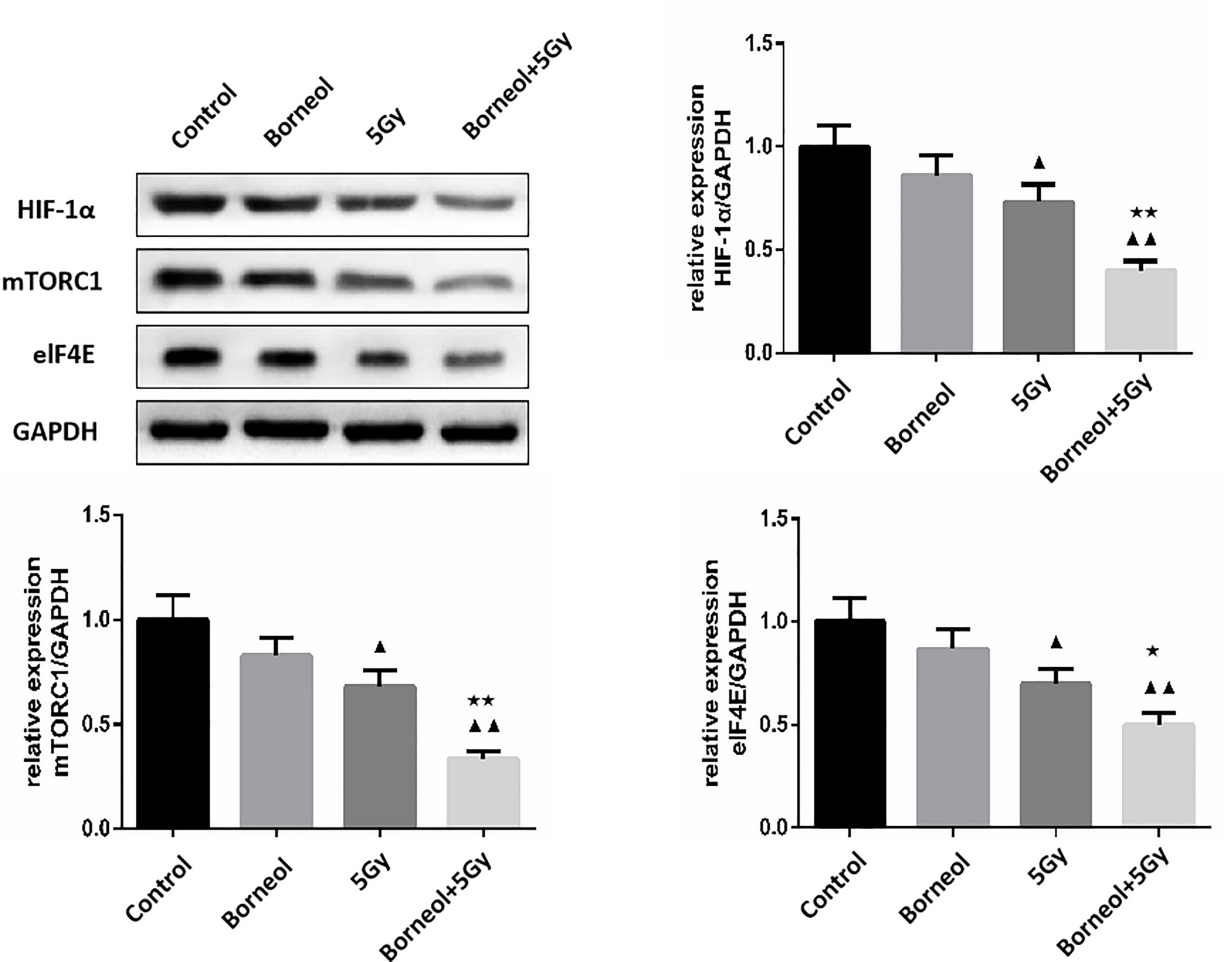
Expression levels of HIF-1α, mTORC1 and eIF4E in human glioma cells after borneol and radiotherapy treatment( $\bar{x}$ ± S,*n*=3). Compared with the control group, ^▲^P <0.05, ^▲▲^P <0.01;Compared with radiotherapy group, ^★^P<0.05, ^★★^P< 0.01.

Furthermore, the combination of borneol and radiation exposure significantly decreased the expression levels of HIF-1α, mTORC1 and eIF4E proteins (**Figures 6**, **7**) in the glioma cells compared to the untreated controls (p < 0.05 or p < 0.01). Compared to radiotherapy alone, the combination treatment had a stronger inhibitory effect (p< 0.05 or p < 0.01).

**Figure 8:**
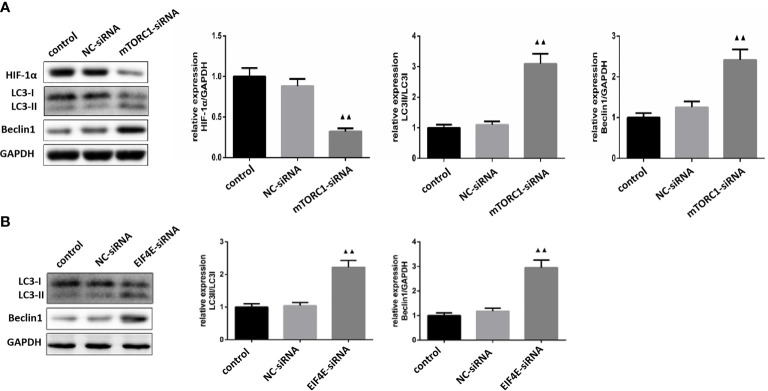
**(A)** Expression levels of HIF-1α, LC3 and Beclin1 in glioma cells silenced by mTOR C1( $\bar{x}$ ± S,*n*=3). **(B)** Expression levels of LC3 and Beclin1 proteins in glioma cells silenced by eIF4E ( $\bar{x}$ ± S,*n*=3). Compared with the normal control group, ^▲▲^ P <0.01.

As shown in **Figure 8A**, mTORC1 knockdown significantly decreased HIF-1α expression in glioma cells (P < 0.01), and increased that of LC3 and Beclin-1 (P <0.01). Furthermore, the expression levels of LC3 and Beclin-1 were also upregulated in the eIF4E-silenced glioma cells (P <0.01)(**Figure 8B**). Taken together, borneol sensitized glioma cells to radiation by targeting the mTORC1/eIF4E/HIF-1α pathway.

## 4 Discussion

Glioblastoma accounts for 50% of all gliomas, and is the most aggressive tumor of the central nervous system in adults, with high recurrence and low cure rates. Despite advances in neurosurgery and the widespread use of chemotherapy and radiotherapy against newly diagnosed GBM, patient survival has improved only marginally. Radiotherapy is the primary treatment strategy for advanced glioma (25, 26), although its outcomes are limited due to hypoxia-induced resistance. HIF-1α is activated in the hypoxic microenvironment of solid tumors, and promotes tumor cell survival by increasing glucose uptake and utilization. In a previous study, we showed that borneol inhibited HIF-1α in primary human glioma cells and rat glioma tissues by targeting the mTORC1/eIF4E pathway, which is also involved in autophagy regulation. Although autophagy generally ensures cell survival and homeostasis by recycling damaged organelles and proteins, excessive autophagy may induce cell death. Therefore, we hypothesized that borneol induces autophagy in glioma cells by targeting the mTORC1/eIF4E signaling pathway, which in turn downregulates HIF-1α and sensitizes the cells to radiotherapy.

HIF-1α activation promotes glioma cell survival, proliferation and metastasis under hypoxic conditions, and is therefore a promising therapeutic target. We found that HIF-1α silencing significantly decreased the proliferation rate of the irradiated glioma cells whereas HIF-1α overexpression had the opposite effect, which confirmed the crucial role of HIF-1α in the response to radiotherapy. Borneol significantly augmented the tumor cell killing effect of radiation *in vitro* and *in vivo*, which coincided with an increase in the number of autophagosomes in the glioma cells/tissues, along with upregulation in LC3 and Beclin1 levels. Beclin1 initiates the process of autophagy by regulating autophagosome formation and maturity. LC3 is a structural component of autophagosomes and a marker of autophagy flux. These results indicate that borneol accelerates autophagy in the irradiated glioma cells, resulting in increased cell death and tumor inhibition. Akt/mTOR inhibitors induce autophagic death in radiation-resistant and radiation-sensitive U87 glioma cell lines, but have no effect on the apoptosis rates and radiosensitivity of glioma cells (27). Consistent with this, borneol significantly downregulated HIF-1α, mTORC1 and eIF4E in the irradiated human glioma primary culture cells, and the inhibitory effect of the combination therapy was stronger compared to radiotherapy alone. Thus, borneol sensitizes glioma cells to radiation by inducing autophagy *via* the inhibition of the mTORC1/eIF4E/HIF-1A pathway. Other studies have shown that inhibition of PI3K/Akt/mTOR pathway and HIF-1α can inhibit the migration and invasion of human glioblastoma U87 cells (28). Consistent with this, silencing of either mTORC1 or eIF4E significantly decreased the levels of HIF-1α in glioma cells, and increased that of LC3 and Beclin-1. This suggests that inhibition of the mTORC1/eIF4E pathway promotes radiosensitivity by inducing autophagy and inactivating HIF-1α.

In conclusion, borneol sensitized glioma cells to radiation by accelerating autophagic cell death through the mTORC1/eIF4E/HIF-1α axis, and should be considered for the treatment of advanced gliomas.

## Data Availability Statement

The raw data supporting the conclusions of this article will be made available by the authors, without undue reservation.

## Ethics Statement

The animal study was reviewed and approved by Animal ethics committee of Zhejiang Cancer Hospital.

## Author Contributions

Conceptualization and methodology by HL and HYZ. Experiments design: QL, LX, CS. Experiments performance: HJZ and MZ. Data analysis: ZW. Writing – Original draft: ZW. Writing – Review & Editing: QL and HL. All authors contributed to the article and approved the submitted version.

## Funding

This work was supported by the 1022 Talent Training Program of Zhejiang Cancer Hospital, National Science of Foundation of China (NO 81502147), Zhejiang Provincial National Science Foundation of China (NO LY20H290003, LGF18H280003, YY18H300008, LYQ20H280001), Zhejiang Medical and Health Science and Technology Project (NO 2020KY477, 2020364926, 2019RC127, 2018KY291,2016KYB038, 2019ZD024), Chinese medicine science and technology plan of Zhejiang Province (NO 2022ZB052, 2021ZB036, 2016ZQ009, 2020ZQ005).

## Conflict of Interest

The authors declare that the research was conducted in the absence of any commercial or financial relationships that could be construed as a potential conflict of interest.

## Publisher’s Note

All claims expressed in this article are solely those of the authors and do not necessarily represent those of their affiliated organizations, or those of the publisher, the editors and the reviewers. Any product that may be evaluated in this article, or claim that may be made by its manufacturer, is not guaranteed or endorsed by the publisher.
